# Relationship between Androgens and Vascular and Placental Function during Pre-eclampsia

**DOI:** 10.3390/cimb46030108

**Published:** 2024-02-21

**Authors:** Lara M. Fernandes, Margarida Lorigo, Elisa Cairrao

**Affiliations:** 1 CICS-UBI, Health Sciences Research Centre, University of Beira Interior, 6200-506 Covilhã, Portugal; fernandeslara287@gmail.com (L.M.F.); margarida.lorigo@gmail.com (M.L.); 2 FCS-UBI, Faculty of Health Sciences, University of Beira Interior, 6200-506 Covilhã, Portugal

**Keywords:** testosterone, androgens, hypertensive disorders of pregnancy, pre-eclampsia, hyperandrogenism, transgender men

## Abstract

Hypertensive disorders of pregnancy (HDP) represent a substantial risk to maternal and fetal health. Emerging evidence suggests an association between testosterone and pre-eclampsia (PE), potentially mediated through androgen receptors (AR). Nevertheless, the mechanism driving this association is yet to be elucidated. On the other hand, reports of transgender men’s pregnancies offer a limited and insightful opportunity to understand the role of high androgen levels in the development of HDP. In this sense, a literature review was performed from a little over 2 decades (1998–2022) to address the association of testosterone levels with the development of HDP. Furthermore, this review addresses the case of transgender men for the first time. The main in vitro outcomes reveal placenta samples with greater AR mRNA expression. Moreover, ex vivo studies show that testosterone-induced vasorelaxation impairment promotes hypertension. Epidemiological data point to greater testosterone levels in blood samples during PE. Studies with transgender men allow us to infer that exogenous testosterone administration can be considered a risk factor for PE and that the administration of testosterone does not affect fetal development. Overall, all studies analyzed suggested that high testosterone levels are associated with PE.

## 1. Introduction

Male sex steroid hormones, such as testosterone (T), are C19-steroids that fall into the androstanes family and are the main molecules responsible for the regulation of numerous biological functions [1,2]. T biosynthesis in males is performed within the testes by Leydig cells through the conversion of cholesterol molecules to the intermediate steroids dehydroepiandrosterone (DHEA) and androstenedione. In target tissues, such as the prostate and seminal vesicles, T can be further metabolized into 5-reduced di-hydroandrogens (5α-dihydrotestosterone (5α-DHT) or 5β-dihydrotestosterone (5β-DHT) metabolites) or in peripheral tissues metabolized to 17β-estradiol [1,2,3]. Despite androgens being known as a male sex hormone, it is also synthesized by females by theca cells and mainly converted into estrogens in neighboring cells of the ovary; in addition, during pregnancy, androgens are also synthesized by the fetoplacental unit [2,3,4].

On target tissues, androgens exert their effect through genomic and non-genomic overlapping mechanisms of action. The genomic or classic mechanism is mediated by the binding of the intracellular androgen receptor (AR) and androgen complex, which regulates the transcription and translation of specific genes and leads to changes in protein synthesis that mediate T effects [5,6]. The genomic pathway requires a longer time frame to achieve the full outcome and is known to mediate secondary male characteristics, libido, and emotions, among other roles [7]. Unlike the genomic pathway, the non-genomic pathway is a rapid pathway mediated by T binding to G-protein-coupled receptors (GPCRs) and ion channels/transporters and the activation of intracellular signaling cascades [8,9].

**Figure 1:**
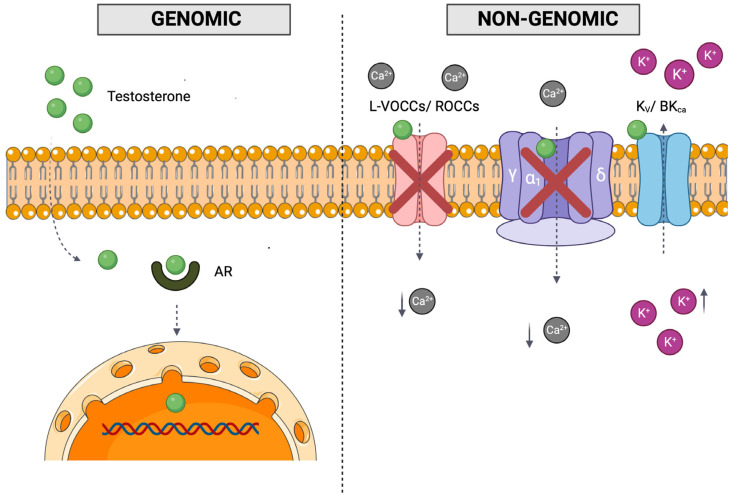
Schematic representation of testosterone vasorelaxation genomic and non-genomic overlapping pathways. The genomic pathway involves the entrance of testosterone into the nucleus, alteration of the transcription and translation process, and the subsequent alteration of protein synthesis, while the non-genomic pathway may involve binding to membrane-associated receptors and/or the alteration of secondary messengers or the modulation of ion channels. Legend: AR—androgen receptor; BK_Ca_—Large Conductance Calcium Activated Potassium Channel; Ca^2+^—calcium; K^+^—potassium; Kv—voltage-gated potassium; L-VOCCs—L-type voltage operated Ca^2+^—Channels; red cross—inhibition; ROCCs—receptor operated Ca^2+^ Channels. Created in Biorender.com. Parts of the figure were drawn by using pictures from Servier Medical Art. Servier Medical Art by Servier is licensed under a Creative Commons Attribution 3.0 Unported License (https://creativecommons.org/licenses/by/3.0/, accessed on 1 September 2023).

It has been established that androgens exert a vasorelaxation effect on the smooth muscle, a rapid effect independent of gene transcription (for review, see [10,11]). However, the molecular mechanism underlying this effect is not clear yet, but the most accepted vasorelaxant action involves changes in intracellular calcium (Ca^2+^) concentration. By blocking the entrance of extracellular Ca^2+^ through the blockage of receptor-operated Ca^2+^ channels (ROCCs) and/or L-type voltage-operated Ca^2+^ channels (L-VOCCs), there is a decrease in Ca^2+^ influx [9]. It was previously demonstrated that T has the same molecular target (α1C subunit of L-VOCCs) as dihydropyridines, which block Ca^2+^ influx in the intracellular space (Figure 1) [12]. In addition, T vasorelaxation activates potassium (K^+^) channels, with voltage-sensitive K^+^ channels (Kv) and large-conductance Ca^2+^-activated K^+^ channels (BKca) being the main ones involved, leading to an increase in K^+^ efflux, although this mechanism might differ depending on the blood vessel [13]. 

Studies on female vasculature have shown that T exerts a relaxant response that is sex-independent (for review, see [10,11]). Despite the scarce research, data suggest that normal T levels in females are beneficial to metabolic function. At the same time, hyperandrogenemia conditions seen in women with polycystic ovary syndrome (PCOS) and congenital adrenal hyperplasia are associated with metabolic disturbances like insulin resistance, metabolic syndrome, and type 2 diabetes mellitus (T2DM) [14,15,16]. Hyperandrogenemia has also been implicated in pathological pregnancies, such as in pre-eclampsia (PE), and in some cases, is associated with the severity of the disease, as reviewed in [17,18]. 

Prior research has scarcely investigated the effect of T level dysregulation on female cardiovascular vasculature, although associations between hyperandrogenemia and infertility and metabolic dysfunctions have been known for a while. Similarly, T’s effects on offspring have been associated with an increased risk of health issues for adult female offspring, but prior investigations have failed to clarify the role of high T levels in hypertensive pregnancies and their involvement in it. Based on this fact, this review aims to analyze the possible implication of T in the pathogenesis of the hypertensive disorders of pregnancy through an extensive review of the literature.

## 2. Approach to the Review

**Figure 2:**
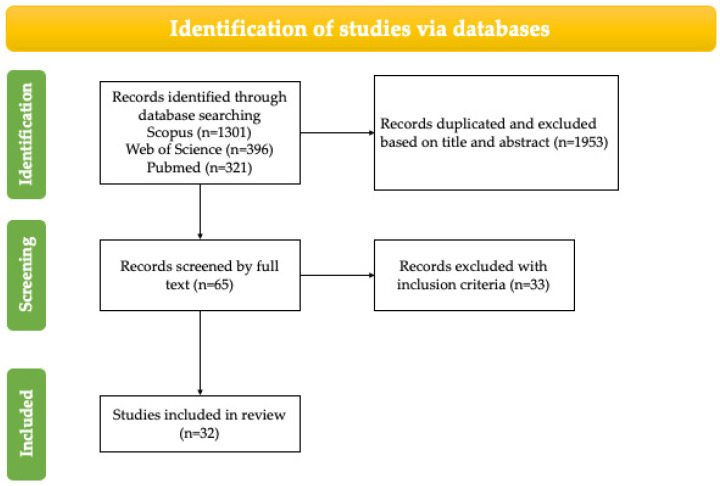
Flowchart for screening and criteria for exclusion of articles.

The latest studies on T’s effects on pre-eclamptic pregnant women’s cardiovascular systems will be presented in the current review (Figure 2). A literature review was carried out for epidemiological and experimental data on the cardiovascular system during pre-eclamptic pathological pregnancy and supported by in vitro and ex vivo studies. Based on a PubMed, Scopus, and Web of Science search, articles published in English and Spanish between 1998 and 2022 were included in this study. Overall, this review discusses the possible association and cause–effect of T on pre-eclamptic pregnant women’s cardiovascular systems. The search terms used included a combination of terms relating to T and pre-eclampsia, and the combinations were as follows: ((androgen [title/abstract] AND pregnancy [title/abstract])) AND ((hypertension [title/abstract]); ((androgen [title/abstract]) AND (gestational hypertension [title/abstract]); (pregnancy-induced hypertension [title/abstract]) AND (androgen [title/abstract]); (pregnancy-induced hypertension [title/abstract]) AND (testosterone [title/abstract]); ((blood pressure [title/abstract]) AND (androgens [title/abstract])) AND (pregnancy [title/abstract]); (androgen [title/abstract]) AND pre-eclampsia [title/abstract]); (androgen [title/abstract] AND (pre-eclampsia [title/abstract]); (maternal androgen [title/abstract]) AND (blood pressure [title/abstract]); ((maternal androgen [title/abstract] OR (maternal testosterone [title/abstract])) AND (hypertension [title/abstract]); (prenatal androgen [title/abstract] AND (hypertension [title/abstract]); (androgen [title/abstract]) AND (prenatal hypertension [title/abstract]); (testosterone [title/abstract]) AND (preeclamptic [title/abstract]); (androgen [title/abstract]) AND (preeclamptic [title/abstract]); (steroid hormone [title/abstract]) AND (pre-eclampsia [title/abstract]); (testosterone [title/abstract]) AND (pre-eclampsia [title/abstract]). Additionally, the bibliographies from selected articles were thoroughly analyzed, and relevant citations were included in this review. Articles that were duplicated, reviews, retrieved, unrelated, and inaccessible were excluded. The inclusion criteria were as follows: (1) the article was original; (2) the study was performed on in vivo animal models, on in vitro models, or using epidemiological data; (3) the study analyzed androgen concentrations associated with PE; (4) the article was written in English. The review was conducted following a weight-of-evidence approach, and the results of the most important studies and those with greater relevance for this review are described below.

## 3. Androgen Receptors in the Placenta and Umbilical Cord

During pregnancy, androgens’ role has been determined in the establishment and maintenance of the pregnancy as well as the start of parturition, hence the elevated levels at birth [4,19,20]. The placenta is an additional source, with its production being a complex process that involves enzymes and other steroids. DHEAS synthesized by the adrenal glands enters the placenta, where is later converted to T and androstenedione (A4) by 17β-hydroxysteroid dehydrogenase (17 β-HSD). Subsequently, T can be converted to 5-reduced metabolites [4]. Previous studies have also reported the expression of cytochrome P450 17A1 (CYP17A1) in trophoblast cells from the human placenta, an enzyme also involved in the biosynthesis of androgens de novo [21]. Moreover, recently, Pereira-de-Morais showed that the umbilical cord vessels, umbilical arteries, and veins of women with pre-eclampsia had different vasoactive responses compared with those of women without alterations [22]. Furthermore, these responses were like those previously obtained by our research group, who observed the response of the umbilical artery after long-term exposure to DHT [23,24,25].

Alongside the placenta, the myometrium synthesizes T, A4, and DHT, and the adrenal maternal gland secretes DHEA, DHEAS, A4, and T. From those maternal sites, the androgens are secreted to the maternal circulation and distributed to peripheral tissues. Additionally, the fetus also produces T and is a source of other androgens, with the levels being sex-dependent. Once biosynthesized, T is converted into DHT in target tissues [19].

Although throughout the pregnancy, an increase in androgens is expected, aberrant levels have been associated with the development of placental dysfunction, often seen in PE; however, its involvement is yet to be demonstrated [17]. 

The androgen receptor (AR) is a nuclear transcription factor that mediates the expression of specific genes and is expressed in numerous target tissues, being more abundant in the male reproductive tract [26]. Hsu et al. detected for the first time the presence of AR in syncytiotrophoblast and stromal cells in human normal and pre-eclamptic placentas in near-term pregnancy [27]. So far, several authors have also demonstrated the presence of AR in the same cells. In the human placenta, ARs are responsible for the regulation of physiological processes such as trophoblast invasion, and differentiation into the syncytiotrophoblast, the outermost layer of the placenta [26]. The role of ARs in PE is not yet fully understood; however, altered AR levels have been observed in placenta samples, and studies have demonstrated that an overexpression of AR might be involved in the pathophysiology of PE, a pregnancy-related condition also characterized by shallow trophoblast invasion that affects proper placentation [27,28,29]. It is suspected that the upregulation of T and AR in the placenta may regulate the expression of specific genes involved in the development of PE.

Sequencing results from the Human Protein Atlas have shown that the TRPV4, OXER1, and ZIP9 protein gene levels of these membrane ARs were highly expressed in trophoblasts. The expression of GPRC6A was also observed in the human placenta, particularly in the syncytiotrophoblast [4]. Computational studies showed T-binding capacity to ZIP9, OXER1, and GPRC6A. The results obtained by D’Arrigo et al. showed that T and DHT had a higher affinity when docked in GPRC6A when compared to ZIP9 and OXER1; however, Kalyvianaki K et al. showed that GPRC6A affinity was lower [30,31]. The different results obtained in both studies are due to the design based on the homology of the receptors, but overall, both studies agree that T can bind to the membrane ARs mentioned above with affinity, and the bound to GPRC6A agrees with the computational study and saturation analysis results obtained by Pi et al. [32]. Regarding GPRC6A expression in pre-eclamptic placentas, it has not been established yet whether there is dysregulation in its expression, but since previous studies have proven that AR expression in pre-eclamptic placentas is altered, it is safe to assume that the same happens to GPRC6A in pre-eclamptic pregnancies, mainly since GPRC6A is involved in the production of interleukin-6 (IL-6), an inflammatory cytokine, previously reported to be elevated in pre-eclampsia [33,34]. Therefore, androgen receptors’ genomic and non-genomic dysregulated pathways might be involved in the development of pre-eclampsia.

## 4. Pre-eclampsia as a Hypertensive Disease of Pregnancy

### 4.1. Overview of HDP

Hypertensive disorders are the most prevalent medical complications during pregnancy and affect about 10% of all pregnancies worldwide, contributing significantly to the rise in maternal, fetal, and neonatal morbidity and mortality [35,36]. In the United States alone, hypertensive disorders of pregnancy (HDP) are among the top six causes of maternal mortality, being responsible for nearly 10% of all maternal deaths [37]. A study conducted in 2005 shows that the prevalence of HDP affects 6% of pregnancies in Portugal, with three quarters of all cases attributed to pregnancy-induced hypertension [38]. Placental abruption, stroke, and multiple organ failure are among the maternal risks included, and as for the fetus, there is intrauterine growth retardation, prematurity, and intrauterine death [39].

During early pregnancy, a decrease in BP occurs, reaching diastolic levels below those observed in non-pregnant women by mid-gestation and returning to normal around the third trimester [40,41]. This drop in BP is due to increased vasodilating agents, such as nitric oxide and prostacyclin [42]. With the progression of pregnancy, cardiac output increases up to 40% to maintain BP, and is also crucial for adequate oxygenation of the fetus and to support the rise in maternal metabolic rate [41]. 

Despite the discrepancies regarding terminologies and definitions, HDP can be classified into the four following categories according to international guidelines [35,36,43]:

- Chronic/pre-existing hypertension. This appears before week 20 (or before preconception).
- Gestational hypertension. This appears mid-pregnancy (after week 20) and returns to normal post-partum.
- Pre-eclampsia–eclampsia. This appears mid-pregnancy along with proteinuria and/or uteroplacental dysfunction and/or some other feature of maternal organ dysfunction.
- Chronic/pre-existing hypertension with superimposed pre-eclampsia–eclampsia. This involves chronic hypertension with signs and symptoms of pre-eclampsia–eclampsia after week 20 of gestation.

According to the “National High Blood Pressure Education Program Working Group on High Blood Pressure in Pregnancy” guidelines, hypertension in pregnancy is systolic blood pressure (SBP) ≥ 140 mmHg and/or diastolic blood pressure (DBP) ≥ 90 mmHg, in two separate measurements [44]. HDP can be further divided into mild and severe with an SBP/DBP, respectively, of 140–159/90–109 mmHg and ≥160/110 mmHg [45].

### 4.2. A Particular Case of Pre-eclampsia

PE is a multiorgan disease process that occurs around the second and third trimesters of pregnancy, and worldwide, its prevalence is 2–8% [46]. In Europe, PE affects 2.8–5.2% of all pregnancies [47]. Often associated with fetal growth restrictions, PE can be life-threatening, being the leading cause of maternal and neonatal morbidity and mortality [48,49]. 

According to the American College of Obstetrics and Gynecology (ACOG), the presence of proteinuria is no longer a requirement for the diagnosis of PE, as long as there is an indication of any end-organ damage; therefore, this HDP can be defined as a mid-pregnancy elevated blood pressure disease with proteinuria (more than 300 mg/24 h), or with the absence of proteinuria but with an elevated creatinine concentration or low platelet count, or in the presence of any end-organ disease, such as the incidence of hematological disorders, renal impairment, hepatic failure, pulmonary edema, or neurological complications [50,51]. 

The etiology is still unknown, but genetic predisposition and immune and environmental factors that may affect the normal development of the placenta have been associated with this disease [52]. Due to its morbidity, it is fundamental to understand its causes to apply effective prevention methods; therefore, multiple theories have been proposed. The renin–angiotensin system (RAS), carbon monoxide, nitric oxide, platelets, and androgens are a few of the mechanisms implicated in the development of this disease. Increased activity of the RAS and alterations in gaseous signaling molecules, like carbon monoxide and nitric oxide, can contribute to increased BP. Alterations in the coagulation process, such as low platelet count, can lead to premature delivery. Lastly, increased androgen levels have been implicated in alterations in blood flow and the formation of the placenta [53,54].

Regarding the role of androgens, we focused on the relationship between elevated maternal T concentration in pregnant women and its possible implication in PE pathogenesis, whether as a causal factor or involvement.

## 5. Effects of Testosterone in Pre-eclampsia

Although in vitro and ex vivo studies often offer a more affordable and quicker approach to research, studies about T’s effects in in vitro and ex vivo models of PE are still scarce. Few authors have used disposable maternal organs, such as the placenta and the umbilical cord of pre-eclamptic women and animal models, to evaluate parameters such as T serum levels and androgen receptor mRNA levels. Vessels from T-treated animals used in in vivo studies have also been extracted for vascular reactivity studies. 

Discarded at birth, the placenta and umbilical cord are endocrine organs responsible for the protection and supply of nutrients and oxygen to the fetus and, therefore, essential for its development [55,56]. The placenta represents a source and target for androgens and has been widely used to measure AR mRNA expression and the umbilical cord as an indicator for prenatal androgen exposure through immunoassays [57]. Therefore, the next sections will present analyses of the effect of T in human and animal studies in in vitro, ex vivo, in vivo, and epidemiological studies. 

### 5.1. Animal Studies

#### 5.1.1. In Vitro Studies

In 2021, Shin et al., to induce PE in pregnant Sprague Dawley rats, divided the rats into five random groups: a control group, a N-nitro-L-arginine methyl ester hydrochloride (L-NAME) group, a catechol-o-methyltransferase inhibitor (COMT-I) group, a Sham group, and a reduced uterine perfusion pressure (RUPP) group. The L-NAME and COMT-I groups were injected daily with 50 mg/kg/day and 2.5 mg/kg/day, respectively, from gestational days (GDs) 10 to 17. The Sham and RUPP groups underwent procedures that reduced uterine blood flow by 40%. By GD18, the animal models were sacrificed, and the placenta was collected to measure T levels through an enzyme-linked immunosorbent assay (ELISA). The results exhibited (Table 1) that T levels were not significantly different between the groups; the control included, however, placental T concentration levels that differed from plasma T levels by 2-fold [58].

#### 5.1.2. Ex Vivo Studies

Several ex vivo studies were performed between 2013 and 2014 by Chinnathambi et al. using arteries from animal rat models of PE to perform vascular reactivity studies through the myograph system. In the first study conducted in 2013, mesenteric arteries were extracted after the rats were sacrificed on gestational day 20 [59]. The T-treated dams’ arteries’ responses to contractile agent phenylephrine (Phe) remained unaffected; however, acetylcholine (ACh) vasorelaxant action was diminished when compared to the control. The authors further assessed the response of ACh in the presence of specific inhibitors to the endothelium nitric oxide synthase (eNOS), endothelium-derived hyperpolarizing factor (EDHF), and prostaglandin I2 (PGI2) pathways. They demonstrated that T treatment affects the ACh vasorelaxant effect through nitric oxide (NO) mediation. Vascular rings treated with losartan (an angiotensin type 1 receptor antagonist) inhibited the angiotensin II (Ang II) vasocontractile effect in both the cases and controls. In the following year, using rat mesenteric endothelium-denuded arteries, the authors found similar responses to cumulative doses of high-potassium solution (KCl) and Phe in the artery segments of controls and cases. In the presence of cumulative doses of Ang II, the vasocontractile response was greater in mesenteric artery segments of T-treated dams, suggesting that T induces hypertension in pregnant rats via angiotensin II receptor type 1 (AGTR1) [60]. In the same year, this time using uterine arteries, the authors used endothelium-intact and endothelium-denuded arteries, which were later subjected to a cumulative dose of ACh and sodium nitroprusside, respectively [61]. The results showed that uterine artery rings from T-treated dams compromise endothelium-dependent relaxation, as well as the NO, EDHF, and prostacyclin endothelium relaxant pathways, with a great impact on the EDHF and prostacyclin pathways, unlike what was previously demonstrated in a different study. The results of the response of the uterine artery segment to the vasoconstrictors’ and vasorelaxants’ effects suggest that its poor vasorelaxant potency and enhanced vasoconstriction may contribute to a decrease in uterine blood flow and therefore the poor delivery of oxygen to the placenta, leading to maternal placental hypoxia. 

In 2018, Perusquía et al., using isolated thoracic aortas from pre-eclampsia-treated and normal pregnant rats previously treated with KCl or Phe, tested the cumulative concentrations (0.1–100 μM) of the androgens DHEA, 5α-DHT, 5β-DHT, and T, and found that T was able to induce vasorelaxation in both PE-treated and normal aortas treated with KCl, and less so in PE-treated aortas with Phe pre-treatment. Thus, the authors proved the vasorelaxant properties of T in the isolated maternal aortas of both PE and normal rat pregnancies [62]. 

In summary (Table 2), vascular reactivity studies on vessels from pre-eclamptic T-treated rat models showed the following: (1) In mesenteric artery rings, T inhibits the NO vasorelaxant pathway, and in the same arteries, induces hypertension via AGTR1. (2) In uterine artery rings, T affects the endothelium-dependent relaxant pathways, with EDHF and PIG2 being the most affected; other authors have demonstrated an imbalance between angiotensin II receptors type 1 and 2, with the latter downregulated when compared to AGTR1, a suggested mechanism that could explain hypertension in PE [63]. (3) Cumulative concentrations of T induce a hypotensive and vasorelaxant effects on the thoracic aortas of pre-eclamptic and normotensive T-treated pregnant rats, respectively.

#### 5.1.3. In Vivo Studies

The etiology of pre-eclampsia is not well understood. Most PE animal model studies focus on the understanding of its pathophysiology or the effects of elevated prenatal androgen exposure on the offspring, hence why so far, to our knowledge, only six in vivo studies aiming to understand the vascular effects of T on pre-eclamptic animal models have been performed.

The earliest in vivo study was performed in 1996 by Liao et al., where a nitric oxide synthase inhibitor, L-NAME, was used to produce similar symptoms to PE [64]. Adult pregnant Harlan Sprague Dawley rats were injected daily with 150 mg/kg/day of L-NAME starting on GD 17 or 18 until delivery on GD 22. Testosterone (0.3 mg/kg/day) and other sex steroid hormones were injected daily after delivery and lasted for 10 days. In addition, L-NAME successfully increased BP steadily and kept it elevated. T considerably decreased BP in post-partum cases, but had the opposite effect on controls.

In 2013, Chinnathambi et al., to mimic the plasma T levels of those observed in PE-like conditions, subcutaneously injected pregnant Sprague Dawley rats with T propionate (0.5 mg/kg/day) from GDs 15 to 19 [59]. The authors measured mean arterial pressure (MAP) continuously and found a steady MAP up until GD 18, observed in both groups; it gradually decreased as the pregnancy progressed, was more elevated in the control group, and increased around delivery on GD 22, like what happens in pregnant women. Furthermore, the authors associate high T levels with an increase in MAP and selective inhibition of the NO-mediated vasorelaxation pathway in pregnant dams.

In the following year, 2014, Chinnathambi et al. mimicked plasma T observed in PE-like conditions, using the same methods as those described above. By GD 20, BP was measured, and as expected, the T propionate-treated group exhibited a higher BP when compared to the control. A set of rats from both the control and T-treated groups who received losartan exhibited similar MAP levels. The research work demonstrated that elevated maternal T levels upregulate AGTR1b mRNA in mesenteric arteries, linking it to increased BP. The authors suggest that increases in the AGTR1-mediated pathway contribute to the maintenance of higher BP in pregnant rats exposed to high T levels, proposing excessive androgen or AGTR1 as potential targets in the treatment of HDP [60]. In the same year, Chinnathambi et al. studied the effects of elevated T levels in the uteruses of T-treated dams and found that T might be a cause of placental hypoxia, which is one of the clinical manifestations of PE [61].

The first and, so far, only study performed in vivo testing androgens’ capacity to attenuate hypertension in a rat model of PE was conducted in 2018 by Perusquía et al. The authors injected female Wistar rats weekly with 20 mg/kg of deoxycorticosterone acetate (DOCA) 5 weeks before and during pregnancy to induce PE. Different doses of androgens (−1.0. to 2.0 log μmol kg^−1^ min^−1^) were administered intravenously via the jugular with ~20 min between each dose. Throughout the administrations, significant reductions in blood pressure and mean arterial pressure were observed with all androgens; the dose–response between T and 5α-DHT was equipotent, and less potent when compared to the two other androgens, dehydroepiandrosterone (DHEA) and 5β-DHT, which were equally equipotent, with DHEA being the one that exhibited the highest antihypertensive response. The data showed the vasorelaxant and hypotensive effects of androgens in both conscious normal and pre-eclamptic rats, also suggesting that a deficiency in androgens production during pregnancy might be a prerequisite to PE development [62].

In 2021, Shin et al., in the same study, used in vivo pre-eclamptic models of the disease [58]. The pregnant rats were divided into five groups: a control group, an L-NAME group, a COMT-I group, a Sham group, and a reduced uterine perfusion pressure (RUPP) group. As described above, the L-NAME and COMT-I groups were injected from GD 10 to GD17 with 50 mg/kg/day and 2.5 mg/kg/day/200 μL, respectively. The Sham and RUPP groups underwent surgical procedures to reduce uterine blood flow by 40%. Among the L-NAME, COMT-I, and RUPP groups, L-NAME was the closest to mimicking critical PE symptoms, hypertension, and proteinuria. Overall, all groups were able to, although through different mechanisms, represent more than one pathological symptom. The authors found differences in T plasma concentration when compared to the placental concentration in in vivo PE models, but other steroid hormones, such as estradiol, pregnenolone, progesterone, and DHEA, were dysregulated in some groups depending on the PE induction method. 

Overall, all methods to induce PE in vivo successfully induced pathological symptoms of pre-eclampsia in animal models (Table 3). Research groups led by Perusquía et al. and Liao et al. effectively demonstrated T’s therapeutic potential in pre-eclamptic in vivo conscious rat models during pregnancy and post-partum, respectively [60,61]. In Liao et al., in a post-partum control, T had the opposite effect and increased blood pressure, but in Perusquía et al., in a pregnant control, T had a vasorelaxant effect. Additionally, Chinnatambi et al.’s in vivo study data were supported by the in vitro and ex vivo studies previously mentioned above. More recently, Shin et al. used three different methods to induce the disease in pregnant rats and found that the L-NAME method was the closest to mimicking pre-eclamptic women’s symptoms [58,59,60,61].

In summary, PE is a complex multifactorial condition that involves several systems in the body. The reproduction of this condition in animal models is difficult and does not always result in phenotypes that faithfully mimic human pre-eclampsia. Moreover, therapeutic interventions in animal models cannot be translated at the full length. Instead, in vivo methods are only able to mimic some of the pathological symptoms, and the results must be analyzed with caution. Moreover, PE occurs spontaneously in humans, while in animal models, it is an induced pathology. Limitations associated with the use of animal models also encompass difficulties in inducing hypertension, challenges in replicating the full extent of the disease, and differences in the placental structure and gestational length [65,66].

### 5.2. Human Studies

#### 5.2.1. In Vitro Studies

It is known that the combination of T and AR triggers a genomic pathway. Hsu et al., hypothesized that the bioactivity between placental steroid hormone receptors and androgen concentration might play a part in the pathogenesis of PE demonstrating a significant increase in AR mRNA expression in the placenta of pre-eclamptic pregnancies when compared to healthy pregnancies [27]. 

Three years later, in 2012, Sathishkumar et al. also discovered greater AR mRNA expression in PE placental samples as well as more visible AR immunostaining; therefore, the author found an association between AR signaling dysregulation, elevated androgen levels, and complications in PE [28]. Placental aromatase levels were also evaluated, and it was found that depending on the fetal sex, they varied. In pre-eclamptic women bearing female fetuses, aromatase expression was higher compared to the control, unlike when the fetus was a male. While in pre-eclamptic pregnancies with female fetuses, the increase in aromatase levels is thought to be a protective mechanism against female fetus virilization, in the male fetus, the decrease in aromatase expression is assumed to contribute to an imbalance in favor of androgens.

In 2020, in China, Lan et al. analyzed the levels of sex steroid receptors in the human placenta of pre-eclamptic and normotensive pregnancies, and found that the two groups had similar mRNA AR levels and distribution in the placenta tissue [67]. Therefore, the authors found no correlation whatsoever with the expression of AR. 

In 2021, Shin et al. used a BeWo human choriocarcinoma-derived cell line to create three different PE in vitro models to access and compare the levels of steroid hormones [58]. To reproduce different PE models, BeWo cells were treated under different conditions. First, one of the groups was submitted to hypoxic conditions known to trigger PE symptoms. The second group was treated with L-NAME; this model inhibits nitric oxide synthesis and can mimic most of the pathological changes associated with this disease. The last model was treated with COMT-I. This drug inhibits the enzyme catechol-o-methyltransferase. A deficiency in this enzyme leads to the development of a PE-like phenotype, leading to conditions such as proteinuria and placental hypoxia. Compared to the control group, the levels of all steroid hormones, including T, were downregulated, and COMT-I exhibited the lowest T levels. Additionally, in the same study, Shin et al. collected placenta tissue from pregnant Sprague Dawley rats and found similar T levels between the groups. 

In summary, greater immunohistochemistry reactivity and mRNA androgen receptor expression in pre-eclamptic placentas were found, suggesting that altered AR signaling might contribute to the pathogenesis of PE, but further research is needed to better understand whether the upregulation in AR signaling is a cause of the development of this condition or a compensatory mechanism (Table 4). However, in 2020, Lan et al. found contradictory data. Both Hsu et al. and Lan et al.’s studies were conducted in Taiwan, and their samples were collected from the same hospital, despite the time lapse difference; therefore, the difference cannot be related to different populations [27,67]. Due to the complexity of the disease, in vitro models of cell lines of PE were unsuccessful in mimicking the upregulated T levels observed in vivo but suggest that the disease affects hormone production.

#### 5.2.2. Epidemiological Studies

Throughout the years, several epidemiological studies have reported an association between elevated T levels and PE diagnosis. The assessment of hypertensive parameters and T levels was performed using blood samples from volunteer pregnant women, which were formerly analyzed by most research groups through RIA; however, there are some exceptions. Overall, the criteria for these studies included being a healthy pregnant woman and having no clinical record until the development of subsequent pre-eclampsia in the study, with no significant difference in body mass index and maternal and gestational age, and, in some studies, some subjects took multivitamins that did not affect the outcomes.

The earliest study was conducted in 1998 in Finland by Laivuori et al., in which blood from women with prior PE was collected and examined 17 years post-delivery [68]. None of the women exhibited clinical signs of hyperandrogenism, but the case study group displayed hyperinsulinemia; overall, the study groups presented similar characteristics. In this study, the levels of serum fT (free testosterone) were found to be higher in women with prior PE compared to the control group, and the total T levels showed no difference between the cases and controls. The SHBG concentration results did not differ between the groups. The authors found that women with a clinical history of PE had elevated T serum levels 17 years post-partum. 

In 1999, Acromite et al., in the United States of America, in a study assessing third-trimester serum androgens levels, showed that primigravid pre-eclamptic women had higher levels of T when compared to normotensive controls, and SHBG concentration showed no significant difference [53]. According to the authors, these findings imply a possible role of androgens in PE pathophysiology, since androgens and PE mediate similar hemodynamic changes.

Additionally, in 2001, in Turkey, Serin et al., to establish a post-partum connection between T levels and PE, collected samples from the third trimester and six weeks after delivery [69]. Third-trimester T and fT levels were found to be significantly higher in the study group when compared to the control. Six weeks after delivery, these levels decreased significantly, and no dissimilarity was found between the two study groups. These findings support the involvement of androgens in PE pathogenesis. 

A year later, in 2002, in the USA, a research team led by Wolf et al. aiming to study early gestation signs to predict PE found no statistical difference between sex steroid levels in PE pregnant women and controls during the first trimester, women who eventually developed PE showed reduced SHBG levels [70], suggesting that first-trimester low SHBG levels may be a marker for PE. In the same year, in Norway, Steier et al.’s study demonstrated that T levels were higher in PE pregnant women, and male-bearing PE pregnancies had the highest levels among cases and controls [71].

In Turkey, in 2003, Fiçicioglu C. and Kutlu measured T levels in the third trimester and divided PE further into severe and mild PE [72]. Total serum T levels showed no statistically significant difference between PE and the control, while fT was significantly higher in the pregnant control group. Furthermore, total T and fT levels were compared between severe and mild PE groups, and both values were found to be higher in the mild PE group, but no significant difference was found for total T. SHBG levels were also measured and found to be higher in the PE group. Around the same time, in Austria, Jireck et al. also discovered elevated serum T concentrations in pre-eclamptic pathological pregnancies compared to a control [73], and in the USA, Troisi et al. also obtained similar results [74]. Additionally, the research group collected umbilical cords from the same subjects, considering the gap in the literature concerning steroid sex hormone levels in the umbilical cord sera of pregnant women. T-cord serum concentrations showed no significant statistical difference, unlike the concentration differences found in the serum of cases and controls of the same subjects [75].

In Turkey, a year later, Basku et al. conducted a prospective study and found that total T and fT levels were considerably higher in the PE group, but no significant difference was found in SHBG levels [76]. In the same year, also in Turkey, Atamer et al. found that total T levels were higher in severe PE, but no significant difference between mild PE and the control was discovered [77]. Both results support the role of androgen in PE pathophysiology, and Atamer et al. go even further, implying that high levels of leptin and T might contribute to endothelial dysfunction in PE.

Carlsen et al., to test the hypothesis that hyperandrogenism is present in early gestation before the onset of PE, conducted a prospective study in 2005 in Norway and Sweden [78]. Maternal androgens and SHBG were measured at weeks 17 and 33 of gestation. Compared to the control, T levels were elevated at weeks 17 and 33 in the cases. In the cases, at week 17, no statistical difference was found between women bearing fetuses of different genders; however, at gestational week 33, only male-bearing women had elevated T levels, and women bearing female fetuses showed no statistical difference with control. The authors identified hyperandrogenism as a marker for PE. During the same year, in Iran, Lou et al. found that fT levels were significantly raised in pre-eclamptic women; however, no significant difference was found in total T levels [79]. The authors identified increased SHBG levels as the cause for reduced fT levels in PE.

In Egypt, in 2006, Salamalekis et al. assessed androgen and SHBG levels in pregnant women during the third semester [54]. Both the fT and total T means were significantly higher in PE, and the results also showed no significant difference in SHBG levels between the groups; therefore, the cause of elevated fT levels could not be attributed to reduced SHBG. In Spain, Vannini et al. found higher T levels in pre-eclamptic women during the third trimester [80].

A year later, in 2007, in Chile, Irribarra et al., looking to clarify whether there is also an association between hyperandrogenemia and chronic/pre-existing hypertension (CH) in pregnant women, designed a study that included normotensive, pre-eclamptic, and chronic hypertensive pregnant women and measured BP, fT, and total T levels [81]. Regarding BP, women with PE and CH exhibited significantly different BP when compared to normotensive women; the highest BP was detected in PE. Regarding T levels, only the total T levels of the pre-eclamptic group showed a significant difference, displaying the highest levels, while the CH group had the lowest, but with no significant difference when compared to the normotensive group. fT levels showed no significant difference between all three groups, but once again, the CH group had the lowest levels. These results show that T levels are not altered in CH, which suggests that this condition is not associated with an increase in T levels, but the authors believe that despite these findings, the measurement of fT levels might not be accurate due to the measurement test chosen. SHBG levels were also measured, and no significant difference was found between the groups.

In the following year, Hsu et al., in Taiwan, found increased mRNA AR expression in pre-eclamptic placentas, and serum T levels measured before delivery also showed that women with PE had elevated T levels [27]. In 2010, Tuuri et al. demonstrated that women with a clinical history of PE showed elevated calculated fT levels only when the follicle-stimulating hormone concentration was below the median, but no significant difference in fT levels was found [82]. In France, Hertig et al. measured T levels in healthy, pre-eclamptic, and severely pre-eclamptic pregnant women with HELLP (Hemolysis, Elevated Liver enzymes, and Low Platelets) syndrome and found similar concentrations in all groups regardless of severity [83].

Still in Finland, a year later, Tuuti et al. measured early-trimester serum T levels to clarify the possible association between hyperandrogenemia and PE [84]. The serum T, SHBG, and, T/SHBG ratio results showed no significant differences that could predict the onset of the disease later; however, despite these findings, the authors believe the methods used to quantify serum T levels were not the most appropriate.

In Iran, during the year 2012, Sharifzadeh et al. compared serum androgen levels in late pregnancy and found greater levels in PE [85]. This time, focusing on the African American population (77% of the study population), Faupel-Badger et al. measured steroid sex hormones in maternal circulation, and although the results were consistent with prior reports from Caucasian populations, the distinction between cases and controls was not enough to reach a statistical difference in T levels. With the same subjects, cord steroid sex hormones were measured and exhibited no difference [86]. In Pakistan, Ashraf et al. found higher serum T levels in PE; additionally, among the cases, those bearing male fetuses exhibited the highest T levels [87].

Three years later, in 2015, in Chile, Perez-Sepulveda et al. once again found raised T levels in PE [88]. The authors also measured placental aromatase expression and, as expected, it was downregulated in PE.

In 2017, this time in China, Shao et al. further divided the PE group into early-onset PE (E-PE), when the clinical signs were present before the 34th week, and late-onset PE (L-PE), after the 34th week [89]. T concentration measurement in E-PE pregnant women had significantly elevated T levels compared to the control. L-PE T concentration was also increased but exhibited no statistical difference compared to the controls.

In 2018, Shin’s research group discovered that T levels measured in the serum and placenta were similar in the cases and controls [90]. In France, Berkane et al. hypothesized that abnormal steroidogenesis occurs long before the diagnosis of PE; however, their results did not differ between the groups [91].

In 2020, also in Bangladesh, another study conducted by Chowdhury et al. found significantly higher fT levels in pre-eclamptic women by the end of gestation when compared to the control [92]. During the same year, in Taiwan, Lan’s research group discovered similar T levels in the umbilical cords of both groups. However, in the same study, the cases exhibited higher serum T levels throughout the pregnancy [67]. In the same year, in Egypt, Ibrahim et al. had similar findings and found an association between PE and women bearing male fetuses [93]. Still in 2020, but this time in Iraq, Al-Maiahy et al. found significantly higher serum T levels in the cases [94].

In 2021, Eckstein et al. in Germany used hair samples to measure androgen levels in pre-eclamptic pregnancies and found similar T levels in the pre-eclamptic and healthy groups [95].

More recently, in 2022, in China, Shen et al. measured serum T levels in pregnant women in the first semester to evaluate whether the serum estradiol-to-T ratio is a biomarker of pre-eclampsia severity. T levels alone showed no significant difference between the groups, although the severe pre-eclamptic group exhibited higher levels [96].

Overall, most of the epidemiological studies agree with greater serum and plasma T level upregulation in PE compared to normotensive pregnancies. Elevated androgen levels seem to have an important role in cervical ripening (dilation of the cervix) at term by regulating cervical collagen fibril organization. Moreover, androgens inhibit myometrial contractility through AR-independent pathways (non-genomic relaxation), which is important for pregnancy reaching term [19]. Although androgen levels gradually increase throughout the third trimester, these levels in PE are 2–3 times higher. Studies have linked high androgen levels to vascular impairment and atypical placentation in PE. Some studies have even measured total and fT serum levels and found that even though in some studies, total T levels might not show significant statistical differences between cases and controls, fT levels are often higher and significantly different in the cases (Table 5). Furthermore, androgen levels in post-partum women with a pre-eclamptic history were assessed, and a significant decrease 6 weeks post-partum was exhibited in one of the studies. In other studies, years post-partum show no significant difference between normotensive women and women with a history of pre-eclamptic pregnancy. The third-trimester SHBG levels measured did not differ between groups, but a study points out that the first trimester had diminished SHBG and suggests a correlation between low first-trimester SHBG levels and the subsequent development of PE. So far, PE has been the only HDP associated with high T levels; however, T is an androgen that is quickly bioconverted by specific enzymes into other sex steroids; dysregulation in the activity of these enzymes alters androgen levels that are secreted into the bloodstream, which may result in abnormal active and nonactive T levels and subsequently affect decidualization, a process linked to the development of PE [3,97,98]. In one of the earliest studies, hyperinsulinemia along with higher fT levels was detected in the study group before PE, which agrees with the metabolic dysfunctions seen in women with hyperandrogenemia. Moreover, T levels in the umbilical cord and placenta exhibited no difference between the groups, but it is known that the placenta is a site for the de novo synthesis of A4, an intermediate for T synthesis, and therefore, despite the low levels exhibited in the sample, the placenta might contribute the increase in T levels. Hair sample analyses also failed to exhibit atypical T levels between cases and controls.

## 6. Therapeutic Potential of Testosterone in the Treatment of Cardiovascular Diseases during Pregnancy

### 6.1. Therapeutic Approach for Pre-eclampsia

Currently, according to international HDP guidelines, the only efficient therapeutic approach for the prevention of preterm PE for women at increased risk of developing the disease is low-dose aspirin (75–100 mg). Such risks include hypertension in previous pregnancies, diabetes, autoimmune diseases, CH, or multiple risk factors for the disease. In Portugal, for pregnant women with more than one risk factor, an intake of 100 mg of aspirin is recommended [99]. Once diagnosed and depending on the severity of the disease, it can be managed with medication to lower blood pressure (antihypertensive drugs), prevent seizures (anticonvulsants), and/or promote fetal lung maturation before delivery (corticosteroids).

According to most medical guidelines, for women with PE, antihypertensive therapy is recommended when SBP > 150–160 mmHg or if DBP > 100–110 mmHg. The most common antihypertensive drugs used as first-line therapies include labetalol, nifedipine, and methyldopa. For the prevention of seizures, magnesium sulfate (MgSO_4_) is the medication of choice, although diazepam can also be used. The use of corticosteroids is advised for women between weeks 24 and 36 of gestation, near delivery in the next days (maximum of 7 days), and the most frequently used corticosteroids are betamethasone and dexamethasone [99]. Continuous monitoring is also extremely crucial for these patients; however, when all the treatments mentioned above fail, the only effective treatment is parturition to prevent maternal and fetal complications due to the progression of the disease, but symptoms can continue even during post-partum [100,101]. In recent years, there has been an increase in evidence associating an increased risk of CVD (cardiovascular diseases) in women with a gestational history of pre-eclampsia when compared to normotensive gestations, hence the importance of the development of novel therapeutic approaches to improve the management of this condition [101]. 

**Figure 3:**
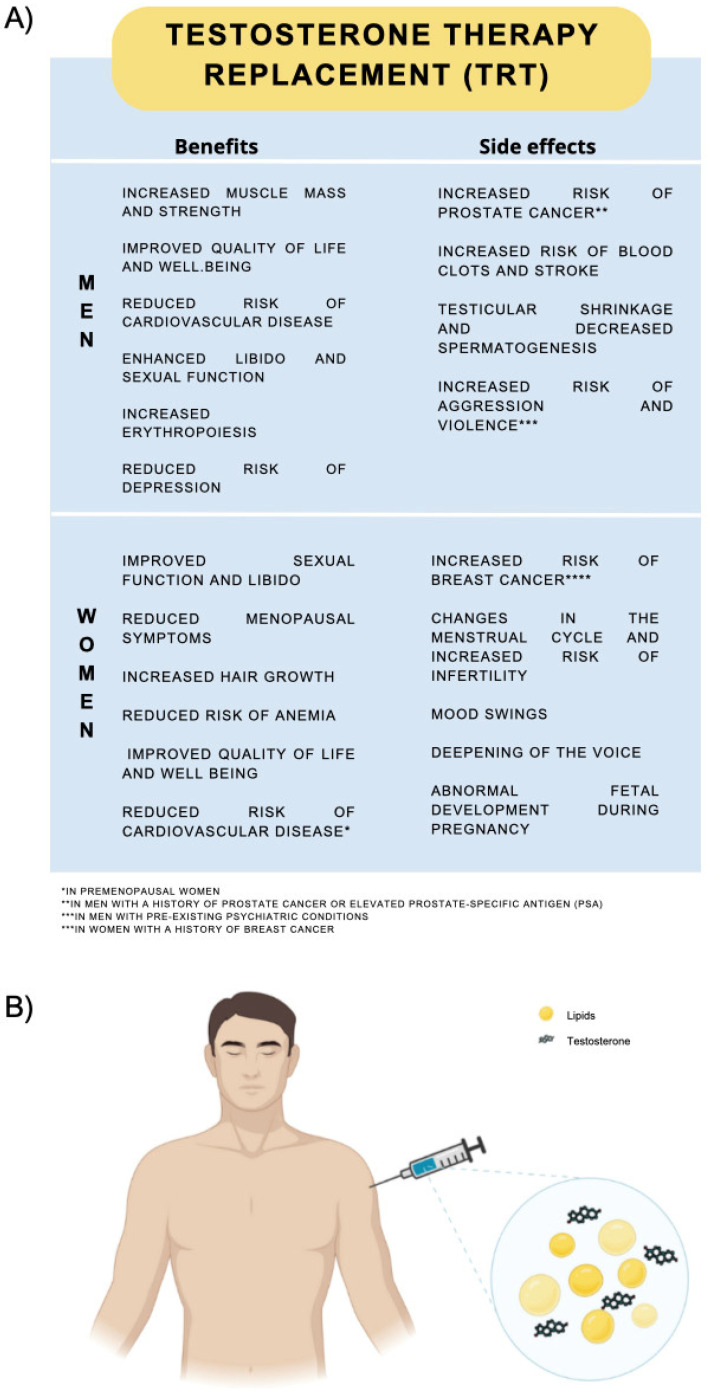
(**A**) Benefits and side-effects of testosterone hormone therapy in men and women. (**B**) Man receiving testosterone via intramuscular injection. Created in Biorender.com (accessed on 22 November 2023).

In women, it has been postulated that hyperandrogenemia raises abdominal adiposity and leads to a subsequent deleterious metabolic profile that is seen as a risk factor for CVD, but that low to normal T levels yield benefits for the skeletal muscle and adipose tissues (Figure 3). Regarding T’s effects on women’s cardiovascular systems, it is still poorly understood, but studies have already demonstrated that T’s action is sex-independent [10,11].

In pregnant women, despite the substantial data available suggesting an association between high T levels and an increased risk of developing PE, these studies are not conclusive evidence of the effect of T on the onset or aggravation of the disease. There is still a huge gap in the literature regarding studies of T’s effects on PE but the few that exist were conducted in animal models and have proven that T has an antihypertensive effect in in vitro and in vivo models, post-partum, and during pregnancy [62,65]. In the study conducted by Perusquía et al., the number of fetuses and fetal weight were further assessed, and the results revealed that the pre-eclamptic rat model exhibited a significantly reduced number of fetuses as well as fetal weight when compared to the controls, but conclusions about the effect of T treatment to treat BP on the fetuses cannot be drawn, since reduced fetal number and fetal weight have been previously reported in other pre-eclamptic animal models of the disease. However, both studies fail to evaluate long-term T dosage’s possible side effects or risks throughout the pregnancy, not only for the mother but also for the fetus, but it has been demonstrated that androgen excess impairs vascular reactivity in FTM (female-to-male) transgender men [102].

### 6.2. A Particular Case for Transgender Men

T is the main hormone used in gender-affirming hormone therapy for transgender men, and despite mixed and puzzling findings regarding its effect on blood pressure, most studies deem T to be effective and safe, but there is a lack of studies in the literature regarding its long-term effects [103,104]. When compared to cisgender women, transgender men present higher exogenous T levels [102,103,104].

**Figure 4:**
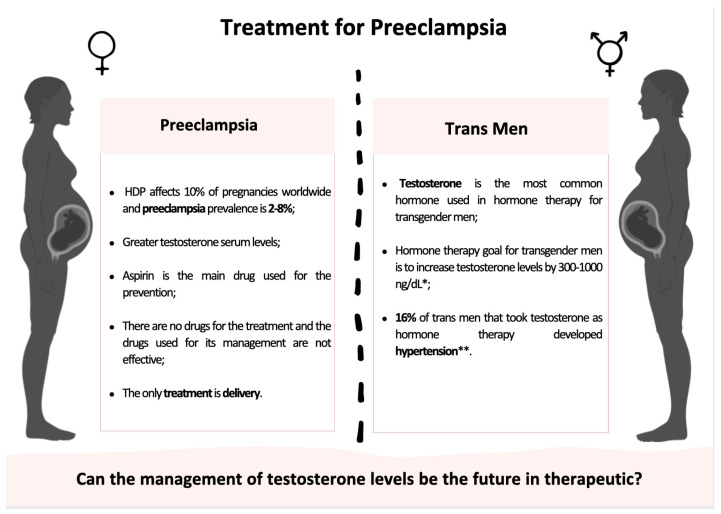
Principal features of the treatment of pre-eclampsia and future application in transgender patients. * and ** Data from [107] and [106], respectively. Created in Biorender.com (accessed on 22 November 2023).

Although they are few, there are reported cases that describe transgender men’s pregnancy with and without assisted reproductive technology after discontinuing hormone therapy, and some even report accidental pregnancies. In a cross-sectional study involving 41 transgender men who had been pregnant, 25 had used T [105]. Regarding the remaining participants, it is not mentioned what they used for the transition process. Among those who reported having an unplanned pregnancy, it is not mentioned how far along they were when they discovered and stopped using hormone therapy medication. Out of the 41 participants in the study, hypertension was reported by 5 participants, 4 of whom had previously used T, but the study does not specify which. Despite studies reporting that transgender men who use T as hormone therapy face the same risks as cisgender women of developing hypertension disorders during pregnancy, the data available are not enough to make assumptions about the matter; however, we can conclude that the percentage of transgender men that developed HDP in this study was far greater than the cisgender population (>16%), but as previously reported, flow-mediated vasorelaxation impairment by T might be the reason [102]. Still, it is urgent to perform new case–control studies where the concentration of T is analyzed before and during pregnancy in transgender men to confirm that increased androgen levels are associated with an increased incidence of HDP (Figure 4). Case–control studies can also give an insight into how greater T levels affect the cardiovascular system during pregnancy as well as into the underlying mechanisms by which T might be involved in the onset of HDP; reliable data on the matter are also important to ensure the development of safe and effective drugs and thus assure a safe and healthy pregnancy for not only transgender men but also cis women, as well as to guarantee the healthy development of the fetus. More recently, in 2022, a case report about an accidental pregnancy in a transgender man receiving T was described [106]. The transgender man in question was unaware of the pregnancy and therefore continued his hormone therapy until the pregnancy was confirmed by week 23. Despite the higher T levels when compared to normal, the patient delivered a healthy female baby who, 3 years later, still showed no signs of abnormal development; it is suspected that she might have autism spectrum disorder, but it is still too early to draw conclusions on whether there might be a cause–consequence case. A long-term evaluation is important to evaluate possible negative outcomes and guarantee her healthy development.

In summary, the current therapeutic approaches to preventing and managing pre-eclampsia are not effective, hence the urgency of developing new drugs. With PE being a multifactorial condition where high T levels and dysregulation in ARs have been proven, the underlying mechanism by which T is involved in the onset and/or aggravation of the disease has not been established yet, but targeting T production and binding to its target receptors might be a potential therapeutic approach.

## 7. Reflective Discussion

The current findings suggest a potential link between high T levels and the development of PE. The underlying mechanism and the complexity of this relationship emphasizes the need for further in-depth investigation due to the severity of this condition. Understanding the complex mechanism behind increased T levels in PE could lead to the identification of possible innovative diagnostic markers and therapeutic targets. Therefore, it could provide insights into early diagnosis and improve maternal and fetal health outcomes. These studies are even more important in the case of transgender men, as it is important to clarify whether the developing fetus is more at risk after hormonal therapy in the progenitor. In our opinion, these studies are very important, as they provide information on the supra-physiological administration of testosterone in pregnancy. However, despite the positive outcomes that could arise from further investigation of T levels in PE, risks such as ethical concerns related to the participants, the potentially invasive procedures for sample collection, the diverse genetic pool, and the complexity of the disease may also pose a challenge in applying possible findings across the worldwide population.

## 8. Conclusions

This review summarizes several studies on T levels in PE and their association with it. An extensive analysis of the literature shows that PE is the only HDP correlated with excessive T levels and AR mRNA upregulation. Overall, elevated T levels were found in PE and correlated with the severity of the disease; however, their implication in PE is still puzzling. Nonetheless, the studies demonstrated that (1) elevated T levels are not a substantial reflection of their total, (2) flow-mediated vasorelaxation might be diminished in PE due to high T levels, as previously observed in transgender men, and (3) AR expression and staining in PE placenta samples were greater, and the physiological processes regulated by it were possibly disturbed.

Pregnancies of transgender men offer an important opportunity to understand the role of the exogenous administration of elevated levels of androgens in the development of hypertensive disorders of pregnancy, its effect on pregnant women, and possible negative outcomes for the fetus. Thus, this review article also intends to explain an increasingly frequent social and medical problem.

## Figures and Tables

**Table 1:** Summary of testosterone levels in in vitro study of a pre-eclamptic animal model.

Animals/Organs/Cells	mRNA Expression	Testosterone Levels (ng/mL)	Assay	Results	Reference
Pregnant Sprague Dawley rats’ placenta	-	4.5 ± 0.6 (L-NAME) vs. 4.7 ± 0.8 (COMT-I) vs. 4.1 ± 0.5 (CONTROL) vs. 4.0 ± 0.5 (Sham) vs. 4.6 ± 1.9 (RUPP) vs. 4.0 ± 0.5 (Sham) Mean ± SD	ELISA	Similar T levels in cases and controls	[58]

**Table 2:** Summaries of vascular effects of testosterone in animal ex vivo studies.

Animals/Organs/Cells	Induction Method	Treatment Administration (μM)	Assay	Results	References
Pregnant Sprague Dawley rats’ mesenteric arteries	Testosterone propionate (0.5 mg/Kg/day) subcutaneously from GD 15–19	—	Vascular reactivity studies (wire myograph)	T affects Ach vasorelaxant effect: increased decline in NO-mediated vasorelaxation in mesenteric arteries of T-treated rats	[59]
Pregnant Sprague Dawley rats’ mesenteric arteries	Testosterone propionate (0.5 mg/Kg/day) subcutaneously from GD 15–19	—	Vascular reactivity studies (Halpern–Mulvaney myograph)	T induces hypertension via AGTR1: increased vasocontractile response in mesenteric arteries from T-treated rats	[60]
Pregnant Sprague Dawley rats’ uterine arteries	Testosterone propionate (0.5 mg/kg/day) subcutaneously from GD 15–19	—	Vascular reactivities studies (multi-myograph)	Decreased uterine blood flow: endothelium-dependent relaxant pathway was compromised in uterine arteries from T-treated rats	[61]
Pregnant Wistar DOCA rats’ thoracic aortas	Deoxycorticosterone acetate (DOCA) (20 mg/kg) subcutaneously weekly	0.1–100	Vascular contractile force measurements (isometric force transducer)	T had hypotensive and antihypertensive effects: T was most potent in KCl pre-treated aortas than its metabolites	[62]

**Table 3:** Summaries of testosterone’s effects in in vivo studies.

Animals/Organs/Cells	Induction Method	Treatment Administration (μM)	Assay	Results	References
Pregnant Harlan Sprague Dawley rats	L-NAME (150 mg/kg/day) subcutaneously from GD 17–18	(0.3 mg/kg/day) for 10 days after delivery	—	T treatment induced a decrease in BP in cases but had an opposite effect on controls	[64]
Pregnant Sprague Dawley rats	Testosterone propionate (0.5 mg/kg/day) subcutaneously from GD 15–19	—	2.1 ± 0.17 vs. 1.0 ± 0.11 Mean ± SEM	Increased T levels by 2-fold in treated dams and higher BP	[59]
Pregnant Sprague Dawley rats	Testosterone propionate (0.5 mg/kg/day) subcutaneously from GD 15–19	—	2.0 ± 0.27 vs. 1.0 ± 0.23 *p* < 0.05 Mean ± SEM	Increased T levels by 2-fold in treated dams and higher BP	[60]
Pregnant Sprague Dawley rats	Testosterone propionate (0.5 mg/kg/day) subcutaneously from GD 15–19	—	—	Increased T levels by 2-fold in treated dams and higher BP	[61]
Pregnant Wistar rats	Deoxycorticosterone acetate (DOCA) (20 mg/kg) subcutaneously weekly	(−1.0. to 2.0 log μmol kg^−1^ min^−1^) was administered intravenously via jugular with ~20 min between each dose	—	T treatment decreased BP in cases, although less when compared to other androgens	[62]

**Table 4:** Summaries of testosterone’s effects in in vitro studies using human organ samples and cell lines.

Animals/Organs/Cells	Induction Method	Treatment Administration (μM)	Assay	Results	References
Human Placenta	36.8 ± 19.0 * vs. 3.7 ± 1.8 (*p* < 0.001)	—	Immunohistochemistry and quantitative RT-PCR	Increased AR mRNA expression in pre-eclamptic placenta samples	[27]
Human Placenta	—	—	Immunofluorescence, Western blot, and quantitative RT-PCR	Increased AR mRNA levels and immunostaining in pre-eclamptic placenta samples	[28]
Human placenta	—	—	Immunohistochemistry, Western blot, and quantitative RT-PCR	Similar AR distribution, mRNA, and protein levels between cases and controls	[67]
BeWo cells	—	0.1 ± 0.0 (Hypoxia) vs. 0.2 ± 0.0 (L-NAME) vs. 0.2 ± 0.0 (COMT-I) vs. 0.3 ± 0.0 (CONTROL)	ELISA	Decreased T levels in pre-eclamptic models when compared to control	[58]

* Ratio between AR gene and a reference gene (Beta-actin).

**Table 5:** Summary of testosterone’s effects in in vitro studies using human organ samples and cell lines.

Biological Matrix	Population/Country	Gestation Age	Testosterone Levels (ng/dL)	Assay	Results	References
Serum	Prior PE (~17 years post-partum) vs. Normal/Finland	—	Free T: 1.6 ± 0.171 vs. 1.166 ± 0.101 Total T: 49 ± 2.884 vs. 40 ± 40 Mean ± SE	RIA	Association between a clinical history of pre-eclampsia and elevated fT serum levels post-partum. Total T levels showed no statistical difference.	[68]
Serum	PE vs. Normal/USA	Third trimester	Free T: 0.5 ± 0.1 vs. 0.3 ± 0.03 Total T: 213.6 ± 25.9 vs. 154.5 ± 14.8 Mean ± SE	RIA	Association between increased T levels in primigravid women and pre-eclampsia	[51]
Serum	PE vs. Normal/Turkey	28–32 and 6 weeks after delivery	28–32 weeks Free T: 0.44 ± 0.12 vs. 0.22 ± 0.13 Total T: 44.1 ± 15.0 vs. 24.3 ± 12.6 6 weeks after delivery Free T: 0.14 ± 0.08 vs. 0.19 ± 0.17 Total T: 24.5 ± 9.4 vs. 23.4 ± 10.9 Mean ± SD	RIA	28–32 weeks: Association between higher T levels and pre-eclampsia 6 weeks after delivery: No difference between cases and controls. Cases exhibited reduced T levels post-partum and similar levels to control	[69]
Serum	PE vs. Normal/USA	10.6 ± 2 Mean ± SD	Free T: 1.025 ± 1.240 vs. 0.944 ± 1.483 Total T: 84 ± 70 vs. 80 ± 98 Mean ± SD	RIA	No association between T levels during first trimester with pre-eclampsia	[70]
Serum	PE vs. Normal/Norway	30–38	Total T: Male 172 ± 22 vs. 82 ± 10 Female 112 ± 12 vs. 72 ± 7.2 Mean ± SEM	RIA	Association between raised T levels in third trimester and pre-eclamptic women. Greater T levels found in pre-eclamptic women bearing male fetuses.	[71]
Serum	PE vs. Normal/Turkey	28–42	Free T (FAI): 5.24 ± 3.57 (Mild PE) vs. 2.72 ± 2.66 (Severe PE) Total T: 274 ± 186 (Mild PE) vs. 210 ± 142 (Severe PE) vs. 219 ± 02 (Control) Mean ± SD	Immulite analyzer and ELISA	Association between higher fT levels and mild pre-eclampsia. No association between total T levels and mild and/or severe pre-eclampsia.	[72]
Serum	PE vs. Normal/Austria	Third trimester (Term)	T: 180 (30–650) vs. 110 (20–220) Median (range)	ELISA	Association between elevated T levels during third trimester (term) and pre-eclampsia	[73]
Serum	PE vs. Normal/USA	Third trimester (Term)	T: 185 (25–920) vs. 150 (38–921) Median (range)	RIA	Association between high T levels in third trimester (term) and pre-eclampsia	[74,75]
Serum	PE vs. Normal/Turkey	Third trimester	Free T: 0.37 ± 0.10 vs. 0.31 ± 0.09 Total T: 257.0 ± 123.8 vs. 136.35 ± 76.2	Free T RIA Total T Immulite 2000, Diagnostic Products Corp., Los Angeles, CA, USA	Association between increased T levels in third trimester and pre-eclampsia	[76]
Serum	PE vs. Normal/Norway and Sweden	17–33	Week 17 Free T (FAI): 0.90 (0.58–1.30) vs. 0.60 (0.45–0.79) Total T: 75 (49–107) vs. 52 (40–69) Week 33 Free T (FAI): 0.83 (0.51–1.36) vs. 0.61 (0.43–0.869) Total T: 87 (61–124) vs. 63 (46–84) Median (IQR)	RIA	Association between increased total T levels in second semester with pre-eclampsia. By week 33, high T levels were restricted to pre-eclamptic women bearing male offspring only.	[78]
Serum	PE vs. Normal/Iran	35.2	Free T: 0.003 ± 0.001 vs. 0.002 ± 0.001 Total T: 1.02 ± 0.10 vs. 1.37 ± 0.019 Mean ± SD	RIA	Association between elevated fT levels in third trimester and pre-eclampsia. Total T levels showed no difference between cases and control.	[79]
Serum	PE vs. Normal/Egypt	28–34	Free T: 0.34 ± 0.05 vs. 0.21 ± 0.02 Total T: 154.4 ± 50 vs. 106.3 ± 39 Mean ± SD	Free T RIA Total T DPC kits and Immulite analyzer (Diagnostic Products Corporation, Los Angeles, CA, USA)	Association between greater T levels third trimester and pre-eclampsia	[54]
Serum	PE vs. Normal/Spain	Third trimester (Term)	Free T: 0.594 ± 0.090 vs. 0.044 ± 0.020 Total T: 152 ± 69 vs. 104 ± 17	RIA	Association between greater T levels at term and pre-eclampsia	[80]
Serum	PE vs. Normal vs. CH/Chile	34–37	Free T: 0.574 ± 0.350 vs. 0.559 ± 0.367 vs. 0.350 ± 0.202 Total T: 150 ± 87 vs. 66 ± 40 vs. 55 ± 43	RIA	Association between higher T in third trimester and pre-eclampsia. Association between lower T levels during the third trimester and chronic hypertension.	[81]
Serum	PE vs. Normal/Taiwan	Third trimester	T: 52 ± 13 vs. 34 ± 11 Mean ± SD	RIA	Association between higher T in third trimester and pre-eclampsia	[27]
Serum	Pre-menopausal women 23–24 years post-delivery with a history of pre-eclampsia/Finland	—	Calculated Free T: 0.761 (0.396–1709) vs. 0.653 (0.272–1.632) Median (range)	LC–MS/MS, API 2000 triple quadrupole mass spectrometer	Association between increased fT calculated levels in women with a history of pre-eclampsia	[82]
Serum	PE vs. Normal/Finland	First trimester 13.7 ± 0.7 vs. 13.7 ± 0.7 (*p* = 0.8) Second semester 19.5 ± 0.8 vs. 19.5 ± 0.7 (*p* = 0.97) Mean ± SD	First trimester T: 69 (55–95) vs. 66 (49–98) Second semester T: 78 (58–104) vs. 75 (52–110) Median (IQR)	LC-MS/MS, API 3000)	No association between T levels during early pregnancy that could predict the onset of the disease	[84]
Serum	PE vs. Normal/Iran	28–34	Free T (pg/mL): 0.128 ± 0.017 vs. 0.074 ± 0.007 Total T: 370 ± 57 vs. 206 ± 24 Mean ± SD	ELISA	Association between greater T levels by the third trimester and pre-eclampsia	[85]
Serum	PE vs. Normal/USA	Third trimester (Term)	T levels: 30000 vs. 21,100 Adjusted mean	RIA	No association between pre-eclampsia and T levels at term	[86]
Serum	PE vs. Normal/Pakistan	≤37	T: Male fetus 4.7 ± 0.35 vs. 1.4 ± 0.14 Female fetus 2.8 ± 0.28 vs. 1.5 ± 0.14 Mean ± SEM	ELISA	Association between greater T levels by third trimester and pre-eclampsia. Highest T levels found among pre-eclamptic women bearing male fetuses	[87]
Serum	PE vs. Normal/Chile	32–36	—	TEXTO-RIA-CT kit	Association of pre-eclampsia and increased T levels in the third trimester	[88]
Serum	PE vs. Normal/Korea	35.6 ± 2.6 vs. 35.2 ± 1.8	—	ELISA	No association of T levels with pre-eclampsia	[90]
Serum	PE vs. Normal/France	24–29	T: 42 [22–83] vs. 32 [16–44] Median [IQR]	GC/MS	No association of T levels in the second trimester with pre-eclampsia	[91]
Serum	PE vs. Normal/Taiwan	35.8 ± 2.2 vs. 38.6 ± 2.8 *p* < 0.001 Mean ± SD	—	Immunoassay systems	Association between elevated T levels throughout the pregnancy and pre-eclampsia	[67]
Serum	PE vs. Normal/Egypt	Third trimester	Total T: 120 ± 40 (Severe PE) vs. 80 ± 20 (Mild PE) vs. 30 ± 10 (Control) Mean ± SD	ELISA	Association between increased T levels and pre-eclampsia. Predominance og male fetuses in pre-eclamptic pregnancies associated with greater T levels.	[87]
Serum	PE vs. Normal/Iraq	≥34	—	ELISA	Association between pre-eclampsia and greater T levels in the third trimester	[94]
Serum	Severe PE vs. Normal/China	First trimester 11–14	—	Chemiluminescent immunoassays	No association between pre-eclampsia and first-trimester T levels	[96]
Plasma	PE vs. Normal/Turkey	34.29 ± 3.478 (Severe PE) vs. 34.73 ± 4.236 (Mild PE) vs. 35.38 ± 3.523 (Control) Mean ± SD	Total T: 45.2 ± 43.9 (Mild PE) vs. 66.9 ± 64.3 (Severe PE) vs. 28.9 ± 29.7 (Control) Mean ± SD	Roche Elecsys 1010 and Modular analytics E 170 (Elecsys Module) immunoassay analyzers	Association between greater t levels in third trimester and severe pre-eclampsia	[77]
Plasma	PE vs. Normal/France	32.5 ± 2.2 (Severe PE with HELLP) vs. 33.7 ± 2.2 (PE) vs. 32 ± 0.7 (Control) Mean ± SEM	T: 46 ± 0.7 (Severe PE with HELLP) vs. 42 ± 17 (Mild PE) vs. 30 ± 0.6 (Control) Mean ± SEM	GC/MS	No association between T levels in third trimester and pre-eclampsia regardless of the severity of the disease	[83]
Plasma	PE vs. Normal/China	33.4 ± 2.3 Third trimester	—	—	Association between elevated T levels in third trimester and early-onset pre-eclampsia	[89]
Plasma	PE vs. Normal/Bangladesh	<37	Free T (nmol/dL): 2.5 ± 0.6 vs. 1.9 ± 0.1 Total T: 0.220 ± 0.030 vs. 0.140 ± 0.020 Median ± 95% CI	Free T ELISA Total T VITROS immunodiagnostic system	Association between higher fT in third trimester and pre-eclampsia. Total T levels showed no significant difference.	[92]
Umbilical cord	PE vs. Normal/USA	Third trimester (Term)	T: 22.0 (6–187) vs. 20.5 (2–166) Median (range)	RIA	No association between T levels by term pregnancy and pre-eclampsia	[74,75]
Umbilical cord	PE vs. Normal/USA	Third trimester (Term)	T:426 vs. 398 Adjusted mean	ELISA	No association between T levels by term pregnancy and pre-eclampsia	[86]
Umbilical cord	PE vs. Normal/Taiwan	Third trimester	—	Immunoassay systems (ADVIA Centaur XP; Siemens USA)	No association between pre-eclampsia and third trimester T levels	[67]
Placenta	PE vs. Normal/Korea	Third trimester	—	ELISA	No association between pre-eclampsia and third-trimester T levels	[90]
Hair sample	PE vs. Normal vs. GDM/Germany	Post-partum	Week 16 T levels (pg/mg): 0.86 ± 0.31 (PE) vs. 0.82 ± 0.37 (Healthy) vs. 1.27 ± 0.75 (GDM) Week 48 T levels (pg/mg): 0.86 ± 0.37 (PE) vs. 0.88 ± 0071 (Healthy) vs. 1.27 ± 0.64 (GDM)	ELISA	No association between T levels and post-partum pre-eclamptic patients	[95]

## Data Availability

Not applicable.
